# Network controllability analysis of intracellular signalling reveals viruses are actively controlling molecular systems

**DOI:** 10.1038/s41598-018-38224-9

**Published:** 2019-02-14

**Authors:** Vandana Ravindran, Jose C. Nacher, Tatsuya Akutsu, Masayuki Ishitsuka, Adrian Osadcenco, V. Sunitha, Ganesh Bagler, Jean-Marc Schwartz, David L. Robertson

**Affiliations:** 1 Dhirubhai Ambani Institute of Information and Communication Technology (DAIICT), Gandhinagar, 382007 India; 2 Department of Information Science, Faculty of Science, Toho University, Funabashi, 274-8510 Japan; 3 Bioinformatics Center, Institute for Chemical Research, Kyoto University, Uji, 611-0011 Japan; 4 Evolution and Genomic Sciences, School of Biological Sciences, University of Manchester, Manchester, M13 9PT UK; 5 Centre for Computational Biology, Indraprastha Institute of Information Technology Delhi (IIIT-Delhi), New Delhi, 110020 India; 6 MRC-University of Glasgow Centre for Virus Research, Glasgow, G61 1QH Scotland UK

## Abstract

In recent years control theory has been applied to biological systems with the aim of identifying the minimum set of molecular interactions that can drive the network to a required state. However, in an intra-cellular network it is unclear how control can be achieved in practice. To address this limitation we use viral infection, specifically human immunodeficiency virus type 1 (HIV-1) and hepatitis C virus (HCV), as a paradigm to model control of an infected cell. Using a large human signalling network comprised of over 6000 human proteins and more than 34000 directed interactions, we compared two states: normal/uninfected and infected. Our network controllability analysis demonstrates how a virus efficiently brings the dynamically organised host system into its control by mostly targeting existing critical control nodes, requiring fewer nodes than in the uninfected network. The lower number of control nodes is presumably to optimise exploitation of specific sub-systems needed for virus replication and/or involved in the host response to infection. Viral infection of the human system also permits discrimination between available network-control models, which demonstrates that the minimum dominating set (MDS) method better accounts for how the biological information and signals are organised during infection by identifying most viral proteins as critical driver nodes compared to the maximum matching (MM) method. Furthermore, the host driver nodes identified by MDS are distributed throughout the pathways enabling effective control of the cell via the high ‘control centrality’ of the viral and targeted host nodes. Our results demonstrate that control theory gives a more complete and dynamic understanding of virus exploitation of the host system when compared with previous analyses limited to static single-state networks.

## Introduction

Virus replication is entirely dependent on the host system they infect. This involves a high degree of virus-host specificity at the molecular level. For example, recognition of receptors on specific cell types by virus molecules is key to cell-entry^1^, and interacting with host molecules is necessary to exploit intra-cellular ‘machinery’ to replicate. To achieve this, virus molecules must interact with many host molecules through a complex network of mostly protein-protein interactions (PPIs)^2^. In turn the host response to infection involves anti-viral factors, and subsequent virus response to host response, leading to a complex entanglement of virus and host interactions^3^.

With the availability of virus-host PPI data sets such as VirusMentha^4^, VirHostNet^5^, HHID^6^ and HCVpro^7^, it is now possible to study viral infection as networks. For instances, human-pathogens protein-protein interactions (PPI) networks have provided a global view of strategies used by different pathogens to subvert human cellular processes and infect them^8,9^, and HIV-human interaction networks have been investigated to provide insights about host-cell subsystems that are perturbed during infection and to investigate approved drug targets^10–12^. It is clear that the specific intra-cellular functions required by a virus to replicate and maintain an infection are the important focus for the virus, and the individual molecular interactions with the host system are just a means to this end^13^.

Linked to networks, graph theory is widely used as a model to describe and visualize perturbation in host cellular systems at the molecular level. For example, the tendency of ‘hubs’ (highly connecting proteins) and high centrality proteins, to be targeted by viruses has been highlighted^8,14,15^. However, in some cases these can be explained by the over-representation of highly-connected molecules in the host function being used^10^. The majority of these studies analyse and visualise the virus-host relationships in static networks, i.e., all of the interactions represented as active, disregarding the temporal and spatial nature of infection. In reality the virus must interact with a complex non-linear dynamic host system. Study of the dynamic nature of infection tends to be limited to specific sub-cellular systems, for example, using logical models in the context of T-cell signalling provided an approach that dynamically modelled the host-viral interactions to identify potential drug targets^16^.

Control theory has emerged as a mathematical framework for understanding how best to control an engineered system, and has largely been applied to study complex dynamic networks and identify ways to control network behaviour^17–21^. The aim is to identify the minimum number of inputs, termed ‘driver’ nodes, that can steer the system from any initial state to any final state in finite time. Past studies of network controllability have identified the driver proteins to be associated with human diseases like cancer^22–26^ and other molecular interaction networks^27–30^.

Viral infection is unique as a system for the study of the applicability of controllability to a natural system, as the virus makes many interactions with the host system and is explicitly exploiting host functions. Past studies have explored the use of control theory in biology. However, in these systems only a few molecules are involved in any instance, e.g., in the case of disease or drug therapy based studies, and so studying all known disease-associated molecules or all drug targets at the same time has little biological meaning^23,31^. In studies where viruses have been investigated explicitly the virus-host interactions have not been included in the network^27,28^, and so control theory has only been partially applied to the system and the effect of inclusion of viral proteins has not been modelled.

HIV-1 and HCV are still a major cause of infections worldwide. An enormous amount of research has been carried out on HIV-1 leading to a very detailed understanding of the virus and use of host systems^3,6^, while studies of HCV have so far identified fewer proteins-host interactions^7,32^. The primary goal of studying these viruses is to obtain a detailed and coherent understanding of infection and viral replication. Critical to this goal is an understanding of viral–host interactions, the host’s immune response and anti-viral mechanisms that play key roles in combating infection.

**Figure 1:**
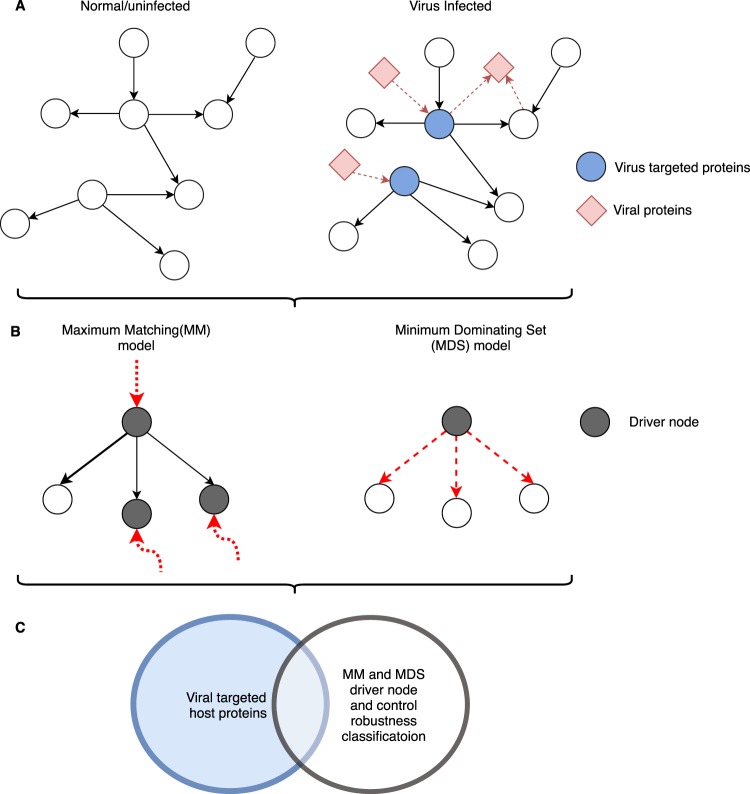
Schematic representation of our network controllability analysis for virus infection. (**A**) Example of normal and infected network. (**B**) Driver node identification using maximum matching (MM) and minimum dominating set (MDS) models. The red dotted arrows indicate inputs to the driver nodes. The bold arrow indicates the matched edge on the maximum matching. The red dashed arrows indicate the nodes controlled by the driver node in MDS. (**C**) Comparison of host proteins that interact with virus and proteins identified as driver nodes.

In this work, we have modelled HIV-1 and HCV host interactions as directed PPI networks both with and without the virus-host interactions included. The signalling network represents biological pathways and is directed. This shows the ordered ‘flow’ of information through the network and so is capturing some of the dynamics of the network. We examine these networks from a controllability perspective and test whether viruses follow the principles of control theory during ‘hijack’ of a host system (Fig. 1). This study of viral infection from a controllability perspective permits, for the first time, testing of the applicability of this mathematical framework to intra-cellular networks and discrimination of available control models (maximum matching versus minimum dominating set^18,19^) using a directed network, consisting of two states: normal/uninfected and infected. Our results lead to novel understanding of the infection mechanism limited to single-state networks, demonstrating the applicability of control theory to the study of infection and validating its use in the study of intra-cellular networks.

## Results

### Identification of driver nodes in the uninfected network

**Figure 2:**
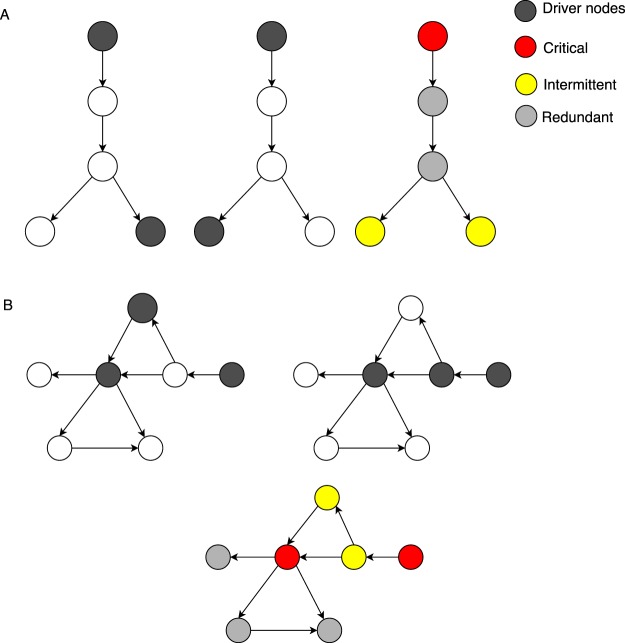
Example of identification and classification of driver node sets into whether a node is always present in these sets (critical driver node), occasionally present (intermittent driver node) or never a driver node (redundant) for the (**A**) maximum matching (MM) and (**B**) minimum dominating set (MDS) models. See key for node designations.

We first identified the driver nodes without the presence of virus in a large directed signalling network comprised of 6339 nodes and 34814 interactions, data from Vinayagam *et al*.^27^. In this network the nodes represent proteins and the edges/links represent directed interactions between them (Supplementary Data File 1). The minimum number of driver nodes (*N*_*D*_) were identified and compared using two established models (i) minimum dominating set (MDS)^19^ and (ii) maximum matching (MM)^18^ (Fig. 2, Methods). This analysis classified 1398 (22%) of the nodes as driver nodes based on the MDS method, compared to 2282 (36%) of them classified by MM (Table 1). The ease with which one can control a network is determined by the minimum number of driver nodes (*N*_*D*_). The lower the number of driver nodes, the easier it is to control the network. Interestingly, the MDS method identified fewer driver nodes compared to MM.

**Table 1:** Classification of minimum dominating set (MDS) and maximum matching (MM) driver nodes.

	MDS model			MM model		
	Normal	Infected		Normal	Infected	
		HIV	Human		HIV	Human
Driver nodes	1398	1232		2282	2264	
Critical	874	12	688	378	1	266
Intermittent	1250	5	1231	3330	2	3443
Redundant	4215	5	4420	2631	19	2630

For a given graph, there can be more than one MM or MDS set and hence there can be multiple minimum driver node sets all with the same size *N*_*D*_. Hence we determined the role of a node as a driver node and classified each node as ‘critical’ if it was present in all driver node sets, ‘intermittent’ if it was present in some driver node set and ‘redundant’ if the node was never part of any driver node set (Fig. 2, Methods). We find 14% of the nodes were critical in MDS, while only 6% of the nodes were classified as critical using MM model. Similarly, the MDS classified more nodes as redundant (67%) compared to MM (42%). On the other hand, the MM model classified most of the nodes as intermittent (53%) compared to MDS (20%) (Table 1). Since the MDS classified fewer nodes as critical and intermittent, this explains why fewer overall driver nodes are required to control the network with MDS. MDS, thus, better reflects optimal control of the signalling network by identifying fewer driver nodes compared to MM.

The driver nodes identified by MM have low degree, specially the critical nodes have zero in-degree, corresponding to receptors on the cell that extracellular molecules (ligands) interact with in order to convey signals to the cell via the signalling pathways. MM only identifies receptors as the driver nodes because this model of control tends to prioritize as drivers those nodes at the beginning of long linear chains, which are controlled externally. The internal nodes of these chains are controlled internally through maximum matched links, whose coupling are consistent with linear response systems as described in the linear mathematical equations shown in Liu *et al*.^18^. Molecular signalling systems, however, are inherently non-linear. Thus, the MM model only detects the initial nodes, such as receptors, as the driver nodes. A result observed in other signalling network studies^27,31^. On the contrary the MDS method does not require structural controllability, here a single integrator node that receives a unique signal from an input link makes the node controllable (Fig. 1B).

In order to assess the roles of proteins in the context of cell signalling, we classified them as either signalling proteins, kinases, receptors and transcription factors^27^. In total the proteins were classified into 1006 signalling proteins, 545 receptors, 366 kinases and 1150 transcription factors (Supplementary Data File 2). We observed that the critical driver nodes obtained through the MDS model played diverse roles in signalling processes and were highly enriched for receptors, consistent with MM (see Supplementary Material, Tables S1 and S2). MDS also showed enrichment for signalling proteins and kinases; the redundant nodes were enriched for transcription factors while the intermittent nodes showed no distinct enrichment. The dynamics underlying biological systems are non-linear as they interact and respond to external and internal cues robustly. In a signalling network, different proteins interact with each other and influence the signal information. Abnormal signal transduction triggers aberrant biological processes that might result in disease. The MDS model not only identified fewer driver nodes, but also identified driver proteins at different levels in the directed network better reflecting the biological reality of both up- and downstream proteins being vital in the control of pathways.

### HIV-1 targeted driver nodes in the uninfected network

Next we looked at the association of virus proteins with driver nodes to identify if they are preferentially targeted by the virus. Out of 6339 proteins in this human directed PPI network, 2529 nodes have been reported to be interacting with HIV-1 from HHID^6^ (See Methods). Of the different MDS driver node classes that interact with an HIV-1 protein we observed that, compared to random samples, 50% of the critical driver nodes were significantly interacting with HIV-1 (Z-score = 6.50) (Table 2), rather than intermittent and redundant nodes, indicating that critical driver nodes are enriched in the virus-host interaction set.

**Table 2:** Classification of MDS driver nodes among the HIV-1 interacting host set. Numbers of observed critical, intermittent or redundant nodes were compared to 1000 random samples.

Node type	Observed	Percentage	Random mean	Z-score	P-value
Critical	438	50.11	349.38	6.5	8.03E-011
Intermittent	489	39.12	498.4	−0.61	0.542
Redundant	1602	38.01	1681.45	−4.41	1.03E-005

To gain more insights into the role of driver nodes and their preference as viral targets, we propose a novel metric for the MDS model: the control centrality (CC) metric that measures the ‘power’ of a node in controlling other nodes or the number of controllable nodes in the network. For MDS, control centrality of a node *v* is *k*_*out*_ + 1, in which *k*_*out*_ denotes the node out-degree. We used this method to compute the control centrality metric for critical, intermittent and redundant nodes (Table 3). For comparison, we averaged this measure and observed that the control centrality of critical driver nodes was more than that of intermittent and redundant nodes. Given that critical driver nodes in the uninfected network were predominantly receptors (45%) and that they have higher power based on CC, this explains their preference as targets by HIV-1 for viral entry and modulation of key host functions.

**Table 3:** Control Centrality (CC) metric of MDS driver nodes.

Average CC			
	Normal	Infected	
		HIV	Human
Critical	16.4	334.67	17.72
Intermittent	6.61	94.8	7.29
Redundant	4.4	48.2	4.77

### Identification of driver nodes in the HIV-1 infected network

Driver nodes were again identified based on the MDS and MM models this time with the inclusion of the virus-host interactions in the directed network (Supplementary Data File 1). For MDS, 1232 (19%) of the nodes were driver nodes, while for MM 2264 (36%) of the nodes were driver nodes (Table 1), results that are approximately comparable to the uninfected network. MDS identified fewer driver nodes compared to MM in the infected networks as well. In the MDS model, 166 nodes lost their driver node status upon infection with HIV, compared to a decrease of only 18 driver nodes for the MM model (Table 1, Supplementary Data File 2)

**Figure 3:**
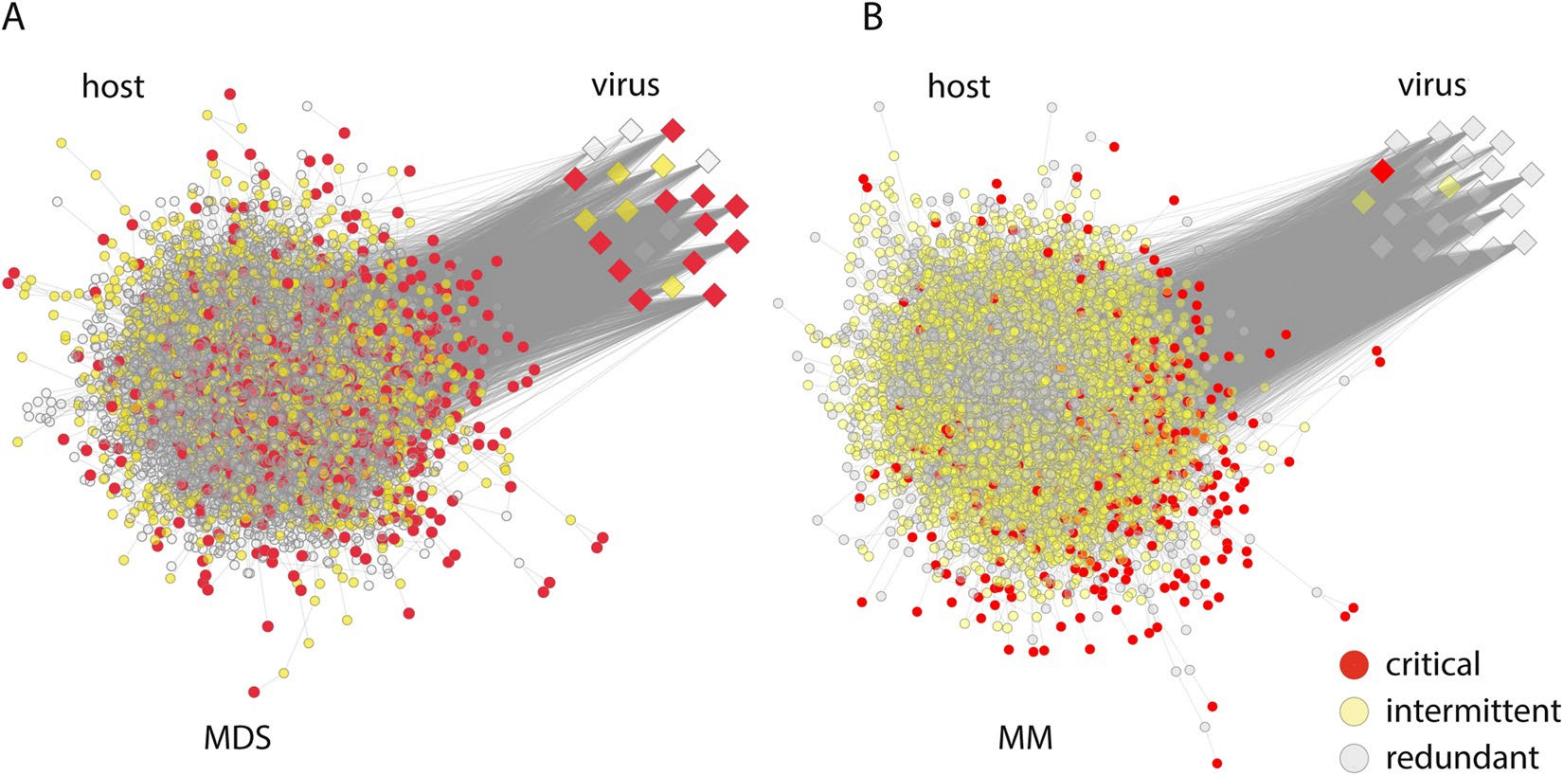
Visualisation of critical, intermittent and redundant driver nodes (see key for designations) for the signalling network infected with HIV-1 for (**A**) the minimum dominating set (MDS) and (**B**) maximum matching (MM) models. Host and viral proteins are shown as circles and squares respectively. Grey lines denote an interaction.

While there is an overall decrease in the number of critical driver nodes identified by both of the models, intermittent and redundant nodes exhibited almost no change for the uninfected and infected states (Table 1). This shows that the virus does not target all of the driver nodes as its not necessarily important for the virus to control the entire network. Importantly, it is only for MDS that the majority of HIV-1 nodes are critical driver nodes (12 of 22), while for MM only 1 of 22 have this property (Table 1). These main 12 HIV-1 proteins are: Nef, gp120, Tat, Pr55(gag), caspid, Rev, gp41, Vpr, Vif, retropepsin, Vpu and p51. In addition the critical nodes identified by MM tend to be peripheral in the network as they correspond to the zero in-degree nodes (Fig. 3). Collectively these results confirm that MDS is matching the viral control of the system much more accurately than MM as they identify the viral proteins as driver nodes along with both up- and downstream host proteins in the signalling network.

These results indicate that inclusion of the virus has increased the controllability of the network, i.e., the virus set of interactions increases the total number of interactions facilitating the hijacking of the cell, and controlling the network more efficiently as fewer driver nodes are required for control. Further, the control centrality analysis for the HIV-1 infected network indicates that HIV-1 molecules are an order of magnitude more ‘powerful’ than host molecules (Table 3).

In the HIV-1 infected network, the MDS model identified 42 nodes as new critical control nodes, while 646 nodes were ‘preserved’ critical nodes, i.e., they retained their driver node status even after HIV-1 infection (Supplementary Material, Figure S1A, and Supplementary Data File 3). Again, this indicates that the controllability model is capturing the biological signal in terms of the virus mechanism of control.

We also looked at those MDS critical driver nodes that HIV-1 interacts with. 438 critical driver nodes were HIV-1 targets in the uninfected network, which reduced to 271 in the infected network (Supplementary Material, Figure S1B). This marked change is presumably due to the inclusion of the HIV-1 host interactions causing many host molecules to lose their critical driver node status. This indicates the presence of virus is fundamentally changing the controllability of the host system. Interestingly, after infection 248 HIV-1 target proteins still preserved their critical driver node status (Supplementary Material, Figure S1B and Supplementary Data File 3). These 248 critical driver nodes are not just important from a control point of view but are also the nodes that are HIV-1 targets (206 of 2087 virus-host interactions) or directed at HIV-1 (109 of 679 host-virus interactions) and could act as potential drug/intervention targets.

We next investigated the biological properties of the preserved critical driver node interacting with HIV-1 by submitting them to the Reactome Pathway database^33^ for enrichment analysis. These critical nodes are enriched for 1341 pathways in the Reactome database. As expected, given the nature of the data, the vast majority were active in signal transduction followed by the immune system. Interestingly, the critical driver nodes seem to play roles in many other cellular subsystems, ranging from cell cycle to developmental biology, disease, programmed cell death and matrix organisation among others, though not as pronounced as in the first two (Table 4). Using the preserved critical proteins, the enriched pathways were analysed to ascertain the top 10 for each set of proteins (Table 4). The list was curated using the FDR rate, p-value for significance, ratio of hit proteins in pathway and similarity percentage between proteins within the enriched systems. Not surprisingly given HIV-1’s life cycle, the top five pathways are specific to the immune system, though most of them also have moderate overlap with RAF/MAP kinase cascades in signal transduction. The next three pathways exclusively deal with signal transduction, followed by programmed cell death, and the last pathway deals with gene expression, transcription in particular. These analyses ascertain the different pathways that the preserved critical driver nodes are involved in and demonstrate the MDS model is a useful method to identify different intra-cellular pathways that HIV-1 interacts with.

**Table 4:** Top 10 Reactome enriched pathways for MDS preserved critical driver proteins between the normal/uninfected and HIV-1 infected network.

Pathway name	Total proteins in pathway	Matching proteins	p-value	FDR
Toll-Like Receptor Cascades	141	14	9.27E-06	1.23E-04
Signalling by Interleukins	460	43	1.15E-14	2.21E-12
CD28 co-simulation	29	6	8.01E-05	5.61E-04
Fc epsilion receptor (FCERI) signalling	405	32	2.96E-09	1.75E-07
Signalling by B Cell Receptor (BCR)	270	23	1.68E-07	5.89E-06
Signalling by NGF	421	45	1.11E-16	5.30E-14
Signalling by PDGF	328	33	3.32E-12	3.52E-10
Signalling by Wnt	230	20	8.29E-07	2.24E-05
Intrinsic Pathways for Apoptosis	41	10	7.61E-08	2.89E-06
SMAD heterotrimer regulates transcription	32	7	1.43E-05	1.57E-04

### Control robustness: before and after infection

To analyse the robustness to control of the signalling network we classified each node into one of the following three categories^27^: (1) ‘indispensable’, i.e., positive control factor, if we have to control more driver nodes in its absence; (2) ‘dispensable’, i.e., negative control factor, if we have to control fewer driver nodes in its absence; and (3) ‘neutral’ control factor if in its absence there is no change in the number of driver nodes to be controlled (Supplementary Data File 2).

Nodes were categorised and compared based on the dispensable classification in both networks using MDS and MM, and the minimum number of the driver nodes (*N*_*D*_) was calculated. Interestingly, MDS classified a much smaller number of nodes as indispensable (503) or dispensable (770) compared to MM (1330 versus 2347 respectively), with twice the number of neutral nodes identified by MDS compared to MM (Table 5). This demonstrates MDS performs more efficiently than MM. A similar trend was seen in the HIV-1 infected network with a smaller number of nodes classified as indispensable (397 human and 11 HIV) or dispensable (719 human and 3 HIV-1) compared to MM (1331 human and 19 HIV-1 proteins verses 2346 human and 1 HIV-1). In addition, on comparing the difference in node characterisation in both states (normal/uninfected and infected), the indispensable nodes reduced by about 20% to 397 for the MDS model but showed no change for the MM model (Table 5). Again these results indicate MDS is a more appropriate model of control for the infected network than MM as this model captured the change in the number of nodes classified as indispensable before and after infection, reflecting the change in control following virus infection.

**Table 5:** Control robustness analysis. Classification of nodes based on its removal between normal/uninfected and HIV-1 infected networks.

	MDS model			MM model		
	Normal	Infected		Normal	Infected	
		HIV	Human		HIV	Human
Indispensable	503	11	397	1330	19	1331
Dispensable	770	3	719	2347	1	2346
Neutral	5066	8	5223	2662	2	2662

Comparing the alternative characterisation of nodes obtained from the MDS model for identifying the overall node control profile, we find that all of the indispensable nodes were critical and all redundant nodes were neutral in both networks; and have deduced a mathematical proof for this property of MDS (See Methods). From a control point of view, indispensable nodes not only determine the ease of control, but also are the driver nodes, since the ability to efficiently control a system is determined by the minimum number of driver nodes. Further, from a biological perspective, these nodes were frequently targeted by HIV-1 (62%) compared to other node classes (see Supplementary Material, Table S3).

### Controllability analysis of HCV human molecular interactome

We also performed controllability analysis using the MDS model for an HCV infected network to validate our findings with another virus. The HCV proteome is comprised of eleven gene products and has a slightly bigger genome than HIV-1. HCV interaction data was obtained from the HCVpro database^7^. A total of 674 interactions were retrieved and curated by matching host proteins with the directed signalling network^34^. Filtering resulted in 11 HCV gene products forming 389 interactions with the PPI network by targeting 325 proteins (Supplementary Data File 1). The direction of the signal flow was assumed by looking at the trend of the interaction type observed for each HCV gene product in the associated literature found on the HCVpro database and comparing the interaction type to the classification system proposed by^15^. The final network consists of 6,350 nodes and 35,202 edges. HCV interacts with 325 proteins out of which 140 were driver nodes and 77 were classified as critical in the uninfected network. In the HCV infected network, 1390 (22%) of the nodes were characterised as driver nodes, and among these driver nodes, 13% were critical, 20% intermittent and 66% redundant (Table S4). All 11 HCV proteins are among the 852 critical driver nodes.

Between uninfected and HCV-infected networks, a higher number of proteins (836 nodes) share critical driver node status, most likely due to the fact that the HCV interacts with fewer proteins (given available data). 16 proteins become critical after HCV infection, while 38 proteins lose their critical node status (Supplementary Material Figure S2A). We analysed 65 preserved critical driver nodes for pathway enrichment (Supplementary Material Figure S2B, Table S5). Many of the top 10 pathways were similar to that obtained for HIV-1, particularly, CD28 co-stimulation, PDGF signalling, signalling by NGF, Fc epsilon receptor signalling and Apoptosis pathway. Regarding signal transduction pathways, the viruses have only NGF and PDGF signalling in common, whilst the remaining signalling pathways seem to be particular to HCV only. Interestingly about 40 proteins are common virus-host interactions shared between HIV-1 and HCV (Supplementary Material, Table S6) and of these 39 are annotated as virus to host and 24 are as host to virus interactions.

### Control models on minimum dominating set

Given that the MDS model more accurately quantifies the viral hijack and control, here we describe the relation between controllability and the minimum dominating set (MDS). Although we only consider directed networks, analogous properties hold for undirected networks if each edge is regarded as two opposite directional edges. We assume in the MDS model that every node in the MDS can assign an arbitrary value to itself and send arbitrary values to all of its outgoing links separately, at any time point. We refer this condition as Condition (**#1**). Intuitively, this condition means that each node can have its own driver signal, which makes control easy. Although this condition might be too strong as a control model of biological systems, it is reasonable for control of artificial systems such as computer networks^19^. Furthermore, several studies showed that many nodes in an MDS are biological important ones^28,30,35^, which suggests that the MDS may capture some important control properties of biological systems. For linear structural controllability in the sense of MM model^18^, the following theorem was proved by modifying the network structure and applying the theory of Liu *et al*.^18^, for undirected networks^19^. The same proof can be extended for directed networks.

#### **Theorem 1**.

*Under Condition* **(#1)***, a network with linear dynamics is structurally controllable by selecting the nodes in an MDS as driver nodes*^19^.

Furthermore, MDS can be applied to controllability analysis of certain kinds of non-linear networks. Here we consider discrete-time systems as a concrete example. Suppose that a given network has *n* nodes, *x*_1_, …, *x*_*n*_. Let *x*_*i*_(*t*) denote the state of node *x*_*i*_ at time *t*. We consider the following dynamics:

$${x}_{i}(t+1)={f}_{i}({x}_{{i}_{1}}(t),\ldots ,{x}_{{i}_{k}}(t)),$$

where ${x}_{{i}_{1}},\ldots ,{x}_{{i}_{k}}$ are input nodes to *x*_*i*_ and *k* depends on *i*. Let *D* be a subset of *R* (*D* ⊆ *R*), where *R* is the set of real numbers. Suppose that each *f*_*i*_ satisfies

$$(\,\forall \,j\,\in [1,k])(\,\forall \,({a}_{1},\ldots ,{a}_{j-1},{a}_{j+1},\ldots ,{a}_{k})\in {R}^{k-1})(\,\forall \,b\in D)(\,\exists \,{a}_{j}\in R)({f}_{i}({a}_{1},\ldots ,{a}_{k})=b),$$

where this condition is referred to as Condition **(#2)**. Various functions satisfy this condition for appropriate domains *D*. For example, every linear function depending on all inputs clearly satisfies this condition for *D* = *R*. For other examples, the following functions satisfy the condition.

$$\begin{array}{rcl}{f}_{i}({x}_{{i}_{1}},\ldots ,{x}_{{i}_{k}}) & = & {\alpha }_{i}{x}_{{i}_{1}}{x}_{{i}_{2}}\cdots {x}_{{i}_{k}}-{\beta }_{i}{x}_{i},\\ {f}_{i}({x}_{{i}_{1}},\ldots ,{x}_{{i}_{k}}) & = & \frac{1}{1+\exp ({a}_{{1}_{1}}{x}_{{i}_{1}}+\cdots +{a}_{{i}_{k}}{x}_{{i}_{k}})},\end{array}$$

for *D* = *R* − {0} and $D=\{x\mathrm{|0} < x < 1\}$, respectively, where *α*_*i*_, *β*_*i*_, and ${a}_{{i}_{j}}$s are non-zero constants and an additional condition of $(\,\forall j)({x}_{{i}_{j}}\ne 0)$ must be satisfied in the former function.

Let *U* be a set of nodes in an MDS. Let *E*′ be the set of edges outgoing from *U*. Under Condition **(#1)**, each *x*_*i*_ ∈ *U* has its own control signal *u*_*i*_(*t*) and each (*x*_*j*_, *x*_*i*_) ∈ *E*′ has its own control signal *u*_*j*,*i*_(*t*). Then, the dynamics of the above system is given by

$${x}_{i}(t+1)=\{\begin{array}{ll}{u}_{i}(t), & \,if\,{x}_{i}\,\in \,U,\\ {f}_{i}({s}_{{i}_{1}}(t),\ldots ,{s}_{{i}_{k}}(t)), & \,{\rm{otherwise}}\,,\end{array}\}$$

where ${s}_{{i}_{j}}(t)={u}_{{i}_{j},i}(t)$ if $({x}_{{i}_{j}},{x}_{i})\in E^{\prime}$, otherwise ${s}_{{i}_{j}}(t)={x}_{{i}_{j}}(t)$. Under this model, the dynamics of the whole system can be represented as **x**(*t* + 1) = *F*(**x**(*t*), **u**(*t*)), where **u**(*t*) is a vector consisting of to *u*_*i*_(*t*)s and ${u}_{{i}_{j},i}(t)$s. Let *d*_*u*_ denote the number of dimensions of **u**(*t*). We will omit ‘(*t*)’ from *u*(*t*) when *t* is not relevant.

#### **Proposition 1**.

*Suppose that the system satisfies Conditions* **(#1)** *and* **(#2)***. Then, for any real vectors* **a** = (*a*_1_, …, *a*_*n*_) ∈ *R*^*n*^ *and* **b** = (*b*_1_, …, *b*_*n*_) ∈ *D*^*n*^, there exists a *d*_*u*_-dimensional real vector **u** such that **b** = *F*(**a**, **u**).

#### *Proof*.

If *x*_*i*_ ∈ *U*, we let *u*_*i*_ = *b*_*i*_. For *x*_*i*_ ∉ *U*, we assume w.l.o.g. that ${x}_{{i}_{1}},\ldots ,{x}_{{i}_{h}}$ are nodes in the MDS, where *h* depends on *i*. From the definition of MDS, *h* > 0 holds for any *x*_*i*_ ∉ *U*. We let ${u}_{{i}_{2},i}=\cdots ={u}_{{i}_{h},i}=0$. Then, from Condition **(#2)**, there exists ${u}_{{i}_{1},i}\in R$ such that

$${b}_{i}={f}_{i}({u}_{{i}_{1},i},\ldots ,{u}_{{i}_{h},i},{a}_{{i}_{h+1}},\ldots ,{a}_{{i}_{k}}).\,\square $$

This proposition means that we can drive the system from state **b** to state **c** in one step by sending adequate signals to MDS nodes and their outgoing edges.

## Discussion

We have compared two network states (uninfected and infected) from a controllability perspective and find HIV-1 and HCV are driver agents in the host system. Importantly, the MDS model more effectively captures the dynamics of viral infection than MM. The performance of MDS validates, for the first time, the applicability of the control theory framework for the study of intra-cellular signalling networks and as a model for studying viral use of host cells. Our results clearly demonstrate the way in which a natural control system (virus exploitation of a host cell) can be used to understand the control of information flow in intra-cellular networks. This hints at the possibility of learning how to synthetically control complex biological systems. In terms of understanding infection, the virus is mainly ‘driving’ the network by exploiting its usual dynamic organisation, i.e., mostly targeting the existing critical driver nodes, with some of the critical driver nodes representing the response to infection.

Interestingly, we demonstrate that indispensable nodes, the positive control factors, are always the MDS critical driver nodes. With the addition of the high-powered viral proteins (as measured by our CC analysis), this control is achieved more efficiently with fewer host molecules acting as critical driver nodes. The MDS proteins, specifically the critical driver nodes, are effectively ‘central’ molecules in terms of information flow in the system^36^ being enriched significantly for proteins that are highly-connected and often multi-functional.

An alternative graph theoretical approach for the study of controllability is the feedback vertex set (FVS) for control^37,38^. For a directed or undirected network, an FVS is a subset of nodes whose removal makes the network acyclic. FVS has been applied to analyses of biological networks. It was shown that singleton attractors in a Boolean network can be enumerated by examining states of nodes in the minimum size FVS^39^. Then, FVS was applied to analysis of Boolean models of metabolic networks^40^. For control of biological systems, Fiedler *et al*.^37^ and Mochizuki *et al*.^38^ showed that selecting FVS as a set of driver nodes, the network can be driven to any statically or periodically steady states under a wide range of dynamical models^37,38^. The FVS method was shown to be useful for controlling real biological networks^41^ as well as to have some advantages over the maximum matching method^42^. Furthermore, the concepts of critical/redundant/intermittent nodes were also applied to the FVS method^43^. However, computation of the minimum size FVS is a well-known NP-hard problem. Although ILP-based methods are known for computing the minimum size FVS^43,44^, it needs huge computation time for large-scale networks^43^, different from the case of MDS. Since our work focused on large-scale PPI networks, we only compared to MM and MDS methods that discuss general controllability to arbitrary states.

In conclusion, using control theory to analyse a complex signalling network in the context of viral infection has proven to be a useful tool for furthering systems biology research. As the virus is mostly interacting with the host systems to replicate itself, involving the up and down regulating of specific host functions, control theory offers an ideal model for the study of this control of information flow, and we believe opens a new discipline of viral control theory. This has the potential to enhance our ability to interfere with infection, for example, by better understanding of the aberrant functions stimulated as a result of infection will be helpful in terms of treating the side-effects/symptoms of infection. Fully understanding the entanglement of viruses with host systems will be the key to limiting their harmful tendencies.

## Methods

### Data procurement and network construction

The human directed signalling network was obtained from Vinayagam *et al*.^27^. It consists of 6,339 proteins and 34,814 interactions. Interaction direction represents potential signal flow between interacting proteins, which was predicted using a Naïve Bayesian Classifier. The classifier assigns a score to each interaction ranging from 0.5 to 1 if there is signal flow, otherwise it assigns a score of 0^34^ (Supplementary Data File 1).

### HIV-1 infected network

HIV-1 interactions were obtained from HHID^6^. A total of 15,230 interactions were retrieved and were further curated by ignoring the number of publications, counting each reaction type only once and selecting only those interactions that had shared nodes with the signalling network. In this network 2,529 human proteins interact with HIV, out of which 308 host proteins are involved in virus replication and infectivity based on siRNA/shRNA-mediated knock-down of human genes in different cell lines. The remaining 2221 human proteins are assigned directions from virus to host or host to virus, giving a total of 5,811 interactions. The directions of the HIV-host interactions were assigned using the method provided by MacPherson *et al*.^13^, where each HHID interaction was assigned a direction based on its interaction type. Direction represents whether the virus protein acts upon the host or vice versa. For example, “Nef activates ACHE” would be given a forward direction as the virus protein acts upon the host, whereas “Nef is activated by ACHE” would be attributed a backward direction, since it is the host protein that activates the virus protein^13^. 2087 and 679 host proteins were involved in virus to host and host to virus interactions, respectively, with 545 having interactions in both directions. The HIV-1 infected network was formed of 6,361 proteins and 40,625 interactions (Supplementary Data File 1).

### Driver node identification

#### Minimum dominating set method

Driver nodes are identified by calculating the minimum dominating set for a given network. For a graph *G*(*V*, *E*), where *V* is set of nodes and *E* is set of edges, a subset $S\subseteq V$ is called dominating set (DS) if every node in *V* is either an element of *S* or is adjacent to an element of *S*. That is for a directed graph, any node *v* ∈ *V*, *v* ∈ *S* holds or there is a node *u* ∈ *S* such that there exists a directed edge (*u*, *v*) ∈ *E* then we say that *v* is dominated by *u*. Then *S* is dominating set if each node in *V* is either in *S* or dominated by some node in *S*. A minimum dominating set (MDS) is a dominating set with the minimum number of nodes. The MDS forms the driver node set. Since the computation of MDS is NP-hard, we used integer linear programming (ILP) to compute the MDS by assigning 0 − 1 variable to each vertex, where 1 is if *v* is part of MDS else 0^19^. A graph can have multiple minimum dominating sets and hence multiple minimum driver node sets with same size *N*_*D*_. So, each node is categorized based on its presence in the driver node set. If a node is always present in all MDS, it is a critical driver node, occasionally present in MDS then it is an ordinary/intermittent driver node and if a node is never part of any MDS then it is a redundant/non-driver node. To address critical controllability in large-scale PPI networks analysed in this study, we used a fast algorithm adapted to directed networks that uses efficient graph reduction using heuristics and mathematical propositions^45,46^. The algorithm for the undirected case was used to analyse large protein interaction networks integrating transcriptome^29^.

#### Maximum matching method

Driver nodes were identified using the controllability package by Liu. *et al*.^18^. The algorithm converts the network into a bipartite graph of two disjoint sets by splitting each node *x*_*i*_ into two nodes (${x}_{i}^{+}$ and ${x}_{i}^{-}$) forming sets of *out* and *in* nodes and placing an directed edge (${x}_{j}^{+}\to {x}_{i}^{-}$) if it was in the original graph. The maximum matching for a digraph was then identified using Hopcroft-Karp algorithm. The unmatched nodes are the driver nodes and the minimum number of driver nodes is denoted by *N*_*D*_. As there could be multiple maximum matchings for a digraph, multiple minimum driver node sets exist with the same size *N*_*D*_. Thus, a node is characterised based on its role as driver node into three categories. If a node is always unmatched then it is a critical driver node, if it sometimes matched and unmatched it is an ordinary intermittent driver nodes while if it is always matched it is a redundant/non-driver node. Thus a node may be never matched, occasionally matched and always matched in the *in* set. Once a matching is found, a matched node say *i* is picked from the *in* set and a node *j* that matched it from the *out* set is identified. While keeping the current matching, node *i* with all its edges is temporarily removed. Starting from node *j* the algorithm checks for an augmenting path that ends to an unmatched node and alternates between unmatched and matched links on that path. If there is no augmenting path the node *i* needs to be always matched and is therefore redundant. If there is an augmenting path, the node *i* is replaceable and hence is intermittent. A node is critical if and only if it has in-degree zero^47^. This procedure is repeated for all nodes in the graph.

### Calculation of control centrality measure of nodes in minimum dominating set

Control centrality is a measure that determines the power of a node to control its sub-systems or other nodes. Mathematically, for a directed network the control centrality of a node *v* is *k*_*out*_ + 1. The reasoning for this count is an MDS driver node controls itself and the outgoing edges independently^19,45^.

### Classification of nodes based on their impact to size of driver nodes set in MDS

The nodes in the network were classified based on their impact on size of drive node set *N*_*D*_ or dominating set (DS). For a network *N* a node was deleted at a time and the MDS was computed for the new network *N*′. If ${N}_{D} > {N}_{D}^{^{\prime} }$ then the node is indispensable. If ${N}_{D} < {N}_{D}^{^{\prime} }$ then dispensable and if ${N}_{D}={N}_{D}^{^{\prime} }$ the node is neutral. We mainly considered undirected networks. However, the proofs can be applied to directed networks too. First, we present an example showing that there exists a node which is critical but is not indispensable. See networks (A) and (B) in Figure S3. Clearly, *v*_1_ is a critical node in the original network of Figure S3A because exclusion of *v*_1_ from MDS increases the size of DS. After removal of *v*_1_, the size of MDS does not change and thus *v*_1_ is neutral. The same properties holds for a directed network Figure S3B.

We also show that in an MDS, every indispensable node is critical and every redundant node is neutral. These properties are different from those obtained by the MM method^27^.

#### **Proposition 1**.

*In MDS, every indispensable node is critical*.

#### *Proof*.

We prove the proposition by contraposition. That is, we show that if *v* is not critical, *v* is not indispensable. Let *G*(*V*, *E*) be an undirected graph. Suppose that *v* ∈ *V*is not a critical node in *G*(*V*, *E*). Then there must exist an MDS $U\subset V$ such that *v* ∉ *U*. Let *G*′(*V*′, *E*′) be the undirected graph obtained by removing v (i.e., deleting v and its connecting edges). Then *U* is a dominating set of *G*′(*V*′, *E*′) because each node *u* ∈ *V* − *v* was dominated by some node *w* ∈ *U*(*w* ≠ *v*) in *G*(*V*, *E*). It means that the MDS size of *G*′(*V*′, *E*′) is not larger than that of *G*(*V*, *E*). Therefore, *v* is not indispensable. $\square$

#### **Proposition 2**.

*In MDS, every redundant node is neutral*.

#### *Proof*.

Let *G*(*V*, *E*) be an undirected graph. First we show that any redundant node is not indispensable. Suppose that *v* is a redundant node in *G*, which means that *v* does not appear in any MDS of *G*. Let *G*′ be the graph obtained by removing *v* from *G*. Let $U\subset V$ be an MDS of *G*. Then *v* ∉ *U* holds, which implies that *U* remains to be a dominating set in *G*′. Therefore, the size of an MDS does increase after removal of *v* and thus *v* is not indispensable. Next we show by contraposition that any redundant node is not dispensable. Let *W* be an MDS of *G*. Suppose that *v* is dispensable. Let *G*′ be the graph obtained by removing *v* from *G*. Since *v* is dispensable, there must exist an MDS $U\subseteq V-\{v\}$ of *G*′ such that $|U|=|W|-1$. Let $U^{\prime} =U\cup \{v\}$. Clearly, *U*′ is an MDS of *G*, which implies that *v* is not redundant. By combining the above two properties, we can see that any redundant node is not indispensable or dispensable. Therefore, the proposition holds. $\square$

### Pathway enrichment

Over-representation of protein in pathways was performed by using the Reactome Pathways database^33^. A p-value cut-off of 0.0001 and minimum matching proteins in pathway of 5 was used as the search parameters. Significant hits were ranked based on the ratio of protein matching, the p-value and False Discovery Rate (FDR), calculated using the Benjamin-Hochberg procedure^48^, which are adjusted p-values used to control the rate of false positives.

## Supplementary information

Supplementary material file

LaTeX Supplementary file

Supplementary data file 1

Supplementary data file 1

Supplementary data file 3
