# Supplementary material for: Network controllability analysis of intracellular signalling reveals viruses are actively controlling molecular systems
Source: Sci Rep. 2019 Feb 14;9:2066. doi: 10.1038/s41598-018-38224-9 (PMC6375943; doi:10.1038/s41598-018-38224-9)
Supplement: Supplementary file 1 — Supplementary material file [file 41598_2018_38224_MOESM1_ESM.docx]

**Supplementary Material**

**Network controllability analysis of intracellular signalling reveals viruses are actively controlling molecular systems**. Vandana Ravindran^1,6^, Jose C Nacher^2^, Tatsuya Akutsu^3^, Masayuki Ishitsuka^2^, Adrian Osadcenco^4^, V Sunitha^1^, Ganesh Bagler^5^, Jean-Marc Schwartz^4^, and David L Robertson^4,6,*^

^1^Dhirubhai Ambani Institute of Information and Communication Technology (DAIICT), Gandhinagar, 382007, India; ^2^Department of Information Science, Faculty of Science, Toho University, Funabashi, 274-8510, Japan; ^3^Bioinformatics Center, Institute for Chemical Research, Kyoto University,Uji 611-0011, Japan; ^4^Evolution and Genomic Sciences, School of Biological Sciences, University of Manchester, Manchester, M13 9PT, UK; ^5^Centre for Computational Biology, Indraprastha Institute of Information Technology Delhi (IIIT-Delhi), New Delhi,110020, India; ^6^MRC-University of Glasgow Centre for Virus Research, Glasgow, G61 1QH, Scotland, UK.

^*^Contact: david.l.robertson@glasgow.ac.uk

|  | **Critical** | | | | **Intermittent** | | | | **Redundant** | | | |
| --- | --- | --- | --- | --- | --- | --- | --- | --- | --- | --- | --- | --- |
|  | Observed | Random mean | Z-  score | P-value | Observed | Random mean | Z-  score | p-value | Observed | Random mean | Z-score | p-value |
| Signaling proteins | 227 | 50.69 | 8.65 | 5.150e-18 | 193 | 198.25 | -0.44 | 0.660 | 586 | 668.90 | -6.11 | 9.963e-10 |
| Receptors | 395 | 74.75 | 41.48 | 0 | 66 | 107.79 | -4.83 | 1.365e-06 | 84 | 362.54 | -26.48 | 1.648e-154 |
| Kinases | 88 | 50.69 | 6.10 | 1.061e-09 | 72 | 72.45 | -0.06 | 0.952 | 206 | 243.17 | -4.20 | 2.669e-05 |
| Transcription factors | 76 | 158.35 | -7.67 | 1.720e-14 | 238 | 227.03 | 0.89 | 0.373 | 836 | 764.73 | 4.81 | 1.509e-06 |

**Table S1.** Cellular characterization of driver nodes based on MDS classification.

|  | **Critical** | | | | **Intermittent** | | | | **Redundant** | | | |
| --- | --- | --- | --- | --- | --- | --- | --- | --- | --- | --- | --- | --- |
|  | Observed | Random mean | Z-score | p-value | Observed | Random mean | Z-score | p-value | Observed | Random mean | Z-score | p-value |
| Signaling proteins | 51 | 60.32 | -1.33 | 0.184 | 388 | 528.76 | -9.69 | 3.325 e-22 | 567 | 418.19 | 10.69 | 1.134 e-26 |
| Receptors | 351 | 32.25 | 62.50 | 0 | 112 | 286.43 | -15.29 | 8.915 e-53 | 82 | 226.79 | -12.89 | 5.124 e-38 |
| Kinases | 29 | 21.62 | 1.70 | 0.089 | 160 | 192.18 | -3.42 | 0.0006 | 177 | 152.2 | 2.70 | 0.007 |
| Transcription factor | 1 | 68.57 | -9.14 | 6.245 e-20 | 367 | 603.15 | -16.09 | 2.998 e-58 | 782 | 477.92 | 20.14 | 3.293 e-90 |

**Table S2.** Cellular characterization of driver nodes based on MM classification.

| **Node type** | **Total** | **HIV-1 Targets (%)** |
| --- | --- | --- |
| Indispensable (503) | 314 | 62.42 |
| Dispensable (770) | 207 | 26.88 |
| Neutral (5066) | 2008 | 39.64 |

**Table S3.** HIV-1 target among the nodes important for ease of control.

| **Driver nodes** | **Critical** | | **Intermittent** | | **Redundant** | |
| --- | --- | --- | --- | --- | --- | --- |
|  | **HCV** | **Human** | **HCV** | **Human** | **HCV** | **Human** |
| 1390 | 11 | 841 | 0 | 1293 | 0 | 4205 |

**Table S4.** Driver node characterization in HCV infected network.

| **Pathway Name** | **Total Proteins in Pathway** | **Matching Proteins** | **p-value** | **FDR** |
| --- | --- | --- | --- | --- |
| Signalling by NGF | 421 | 18 | 4.81E-10 | 8.92E-08 |
| CD28 co-stimulation | 29 | 7 | 1.97E-09 | 2.05E-07 |
| Signalling by PDGF | 328 | 15 | 7.58E-09 | 5.23E-07 |
| GAB1 signalosome | 115 | 10 | 9.56E-09 | 5.93E-07 |
| DAP12 signalling | 308 | 14 | 2.84E-08 | 1.25E-06 |
| Intrinsic Pathway for Apoptosis | 41 | 6 | 5.25E-07 | 1.47E-05 |
| Signalling by Interleukins | 460 | 15 | 6.11E-07 | 1.53E-05 |
| Fc epsilon receptor signalling | 405 | 14 | 7.89E-07 | 1.89E-05 |
| Rap1 signalling | 16 | 4 | 5.99E-06 | 1.02E-04 |
| Regulation of TP53 Activity | 145 | 7 | 8.10E-05 | 9.51E-04 |

**Table S5.** Pathway enrichment analysis of preserved critical driver nodes in the HCV network.

| **Name** | **MDS** | **Category** | **Essential** | **HIV(RNAi)** | **Druggable** |
| --- | --- | --- | --- | --- | --- |
| STAT3 | TRUE | critical | Yes | - | - |
| LYN | TRUE | critical | Yes | - | Yes |
| CSNK2A1 | TRUE | critical | Yes | - | Yes |
| CDKN1A | TRUE | critical | Yes | - | - |
| TGFBR1 | TRUE | critical | Yes | - | Yes |
| MLLT4 | TRUE | critical | - | - | - |
| CTSB | TRUE | critical | Yes | - | Yes |
| VCAN | TRUE | critical | Yes | - | - |
| AKT1 | TRUE | critical | Yes | Yes | Yes |
| RAF1 | TRUE | critical | Yes | - | Yes |
| ACTN1 | TRUE | critical | - | - | - |
| LCK | TRUE | critical | Yes | - | Yes |
| JAK1 | TRUE | critical | Yes | Yes | Yes |
| CSNK2B | TRUE | critical | - | - | - |
| TBP | TRUE | critical | Yes | - | - |
| GRB2 | TRUE | critical | Yes | - | - |
| GSK3B | TRUE | critical | Yes | - | Yes |
| SRC | TRUE | critical | Yes | - | Yes |
| EEF1A1 | TRUE | critical | Yes | - | - |
| JUN | TRUE | critical | Yes | - | - |
| RXRA | TRUE | critical | - | - | Yes |
| YWHAZ | TRUE | critical | - | - | - |
| VTN | TRUE | critical | - | - | - |
| CASP8 | TRUE | critical | Yes | - | Yes |
| HCK | TRUE | critical | Yes | - | Yes |
| SMAD3 | TRUE | critical | Yes | - | - |
| PLSCR1 | TRUE | critical | - | - | - |
| PPARA | TRUE | critical | - | - | Yes |
| APOA1 | TRUE | critical | Yes | - | - |
| ITGB1 | TRUE | critical | Yes | - | Yes |
| HSPA5 | TRUE | critical | Yes | - | - |
| TP53 | TRUE | critical | Yes | - | Yes |
| RAB5A | TRUE | critical | - | - | - |
| EP300 | TRUE | critical | Yes | Yes | - |
| PRKACA | TRUE | critical | Yes | - | Yes |
| CREBBP | TRUE | critical | - | - | - |
| TRAF2 | TRUE | critical | - | - | - |
| TNFRSF1A | TRUE | critical | Yes | - | - |
| CD81 | TRUE | critical | - | - | - |
| HLA-A | TRUE | critical | - | - | - |

**Table S6.** Critical driver nodes common among the HIV-1 and HCV networks.


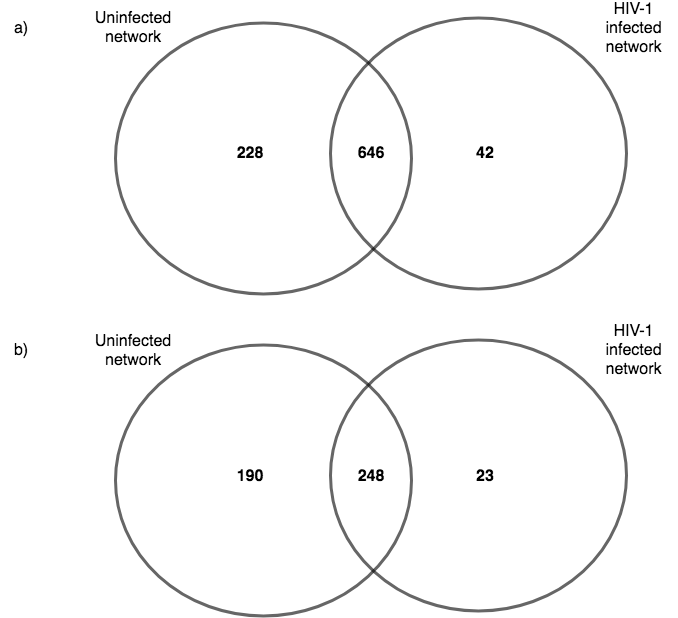


**Figure S1**. Comparison of MDS critical driver nodes in uninfected and HIV-1 infected networks. Venn diagram representing the comparison (a) among critical driver nodes in uninfected and HIV-1 infected networks and (b) of HIV-1 targeted critical driver nodes in uninfected and HIV-1 infected networks.

**
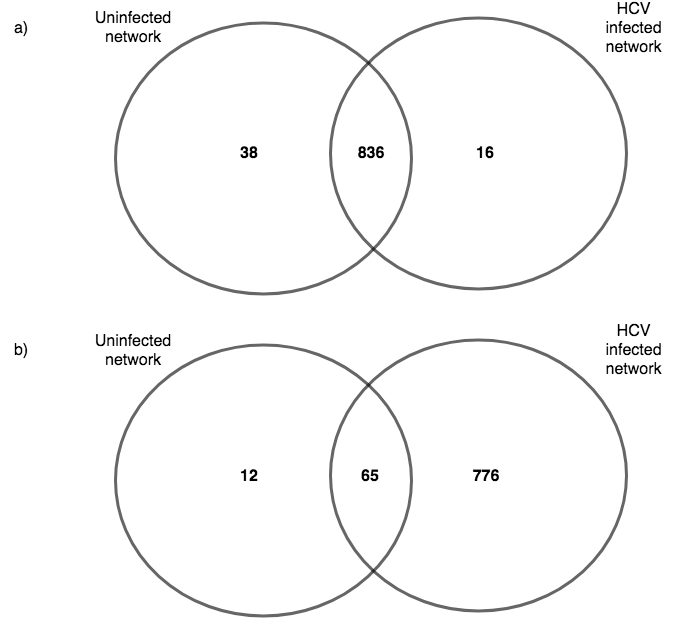
**

**Figure S2.** MDS critical driver nodes in uninfected and HCV infected networks. Venn diagram representing the comparison (a) among critical driver nodes in uninfected and HCV-infected networks and (b) of HCV targeted critical driver nodes in uninfected and HCV-infected networks.

**
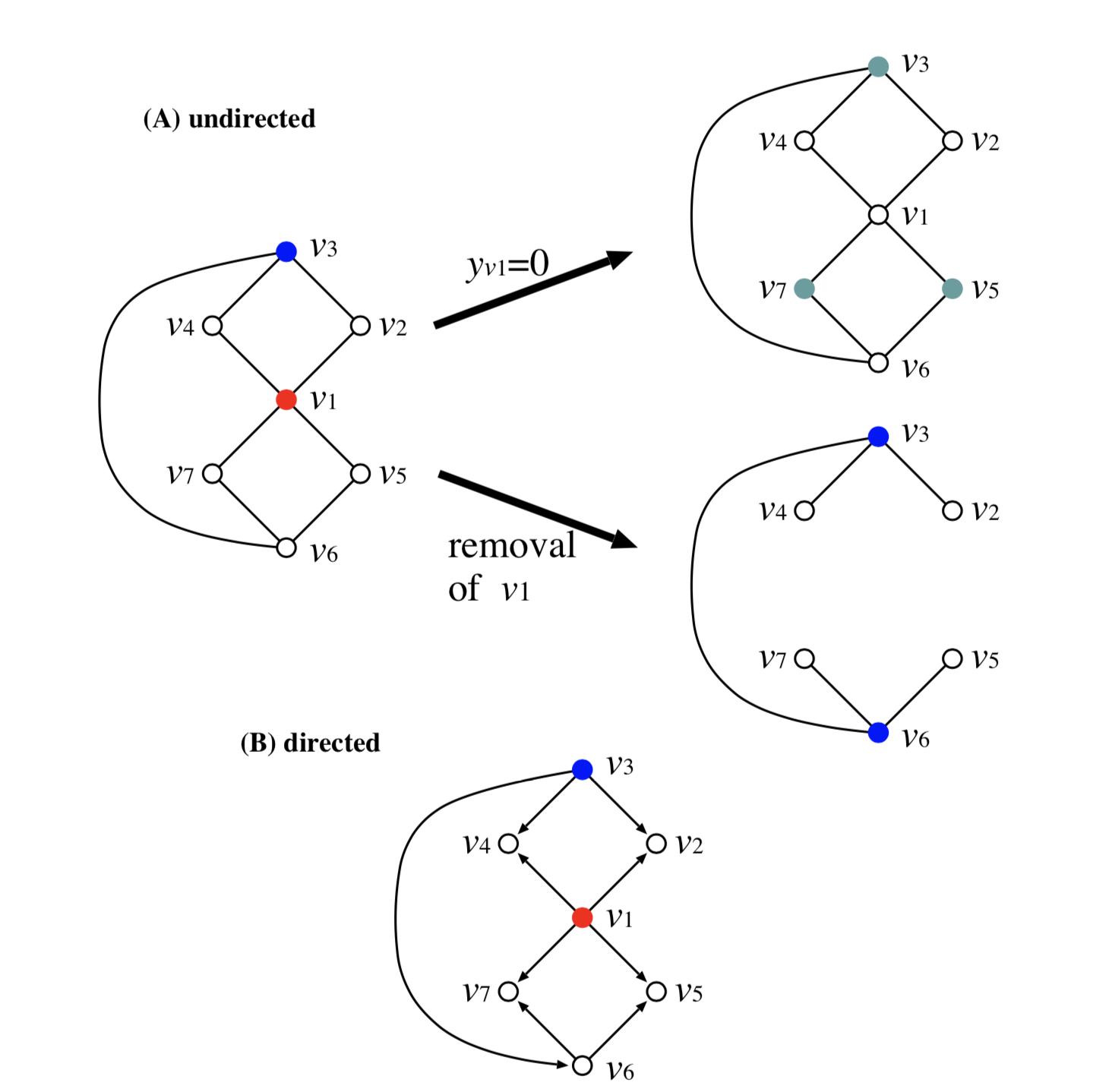
**

**Figure S3.** Examples of MDSs in (A) undirected and (B) directed networks. Nodes in an MDS are shown by red and blue circles, where critical nodes are shown in red color. It is seen from (A) that $v_{1}$ is critical but neutral. In (B), $v_{1}$ is critical but neutral too.
