# Supplementary figures and images for: Network controllability analysis of intracellular signalling reveals viruses are actively controlling molecular systems
Source: Sci Rep. 2019 Feb 14;9:2066. doi: 10.1038/s41598-018-38224-9 (PMC6375943; doi:10.1038/s41598-018-38224-9)

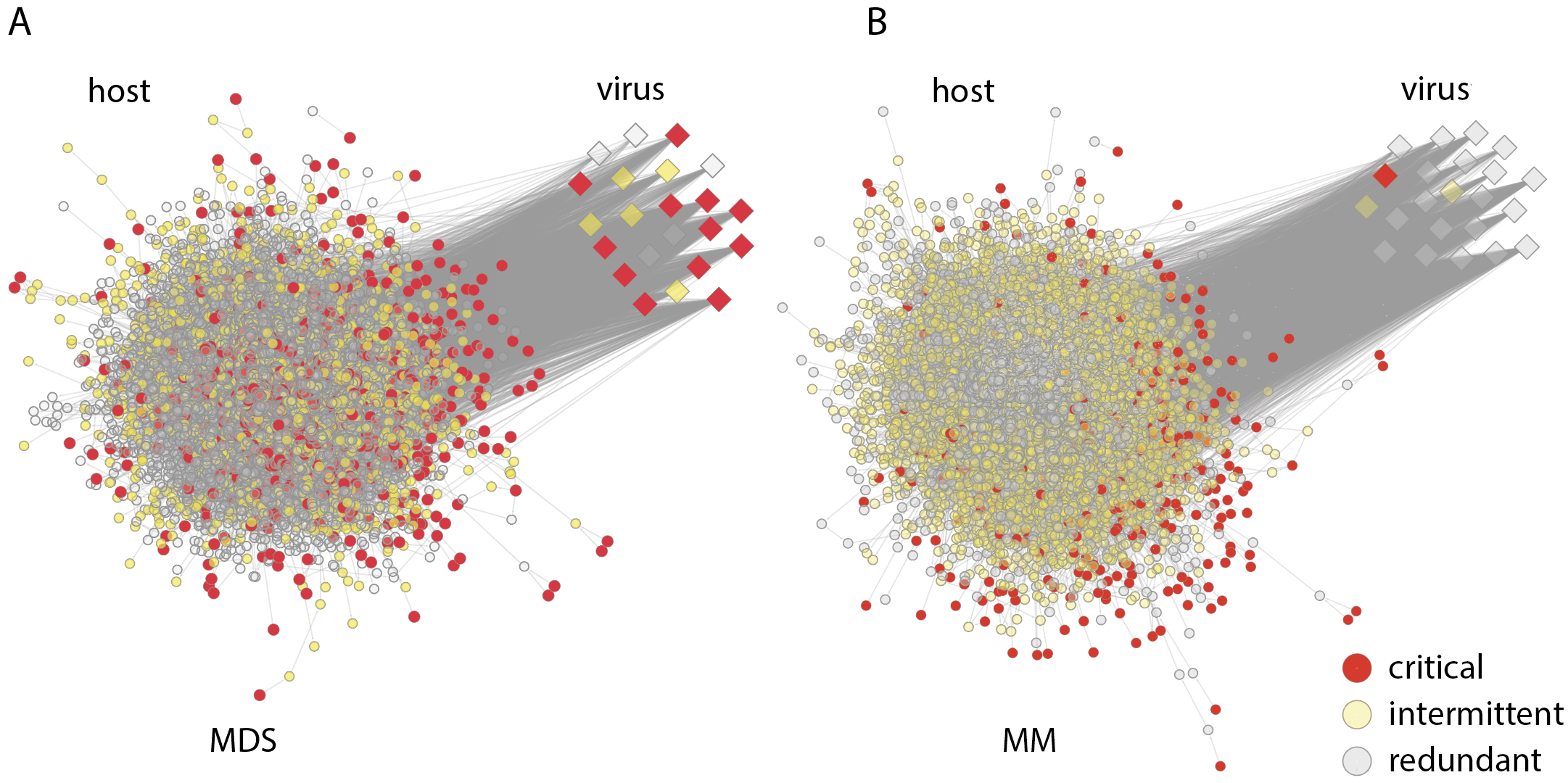

Supplement: Supplementary file 2 — LaTeX Supplementary file [file 41598_2018_38224_MOESM2_ESM.zip › Figure_3.png]

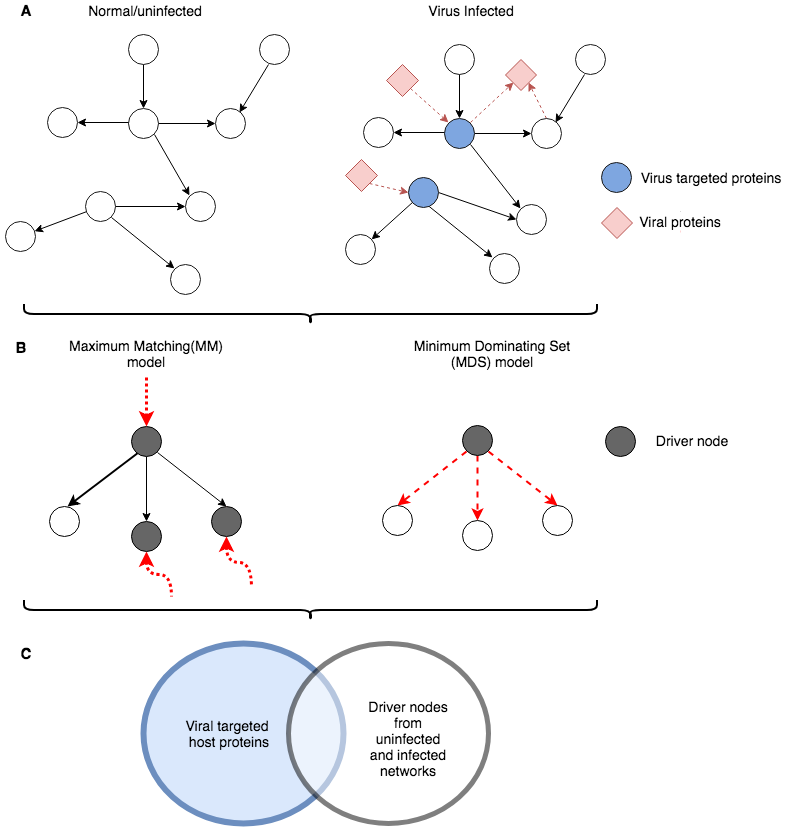

Supplement: Supplementary file 2 — LaTeX Supplementary file [file 41598_2018_38224_MOESM2_ESM.zip › figure_1.png]

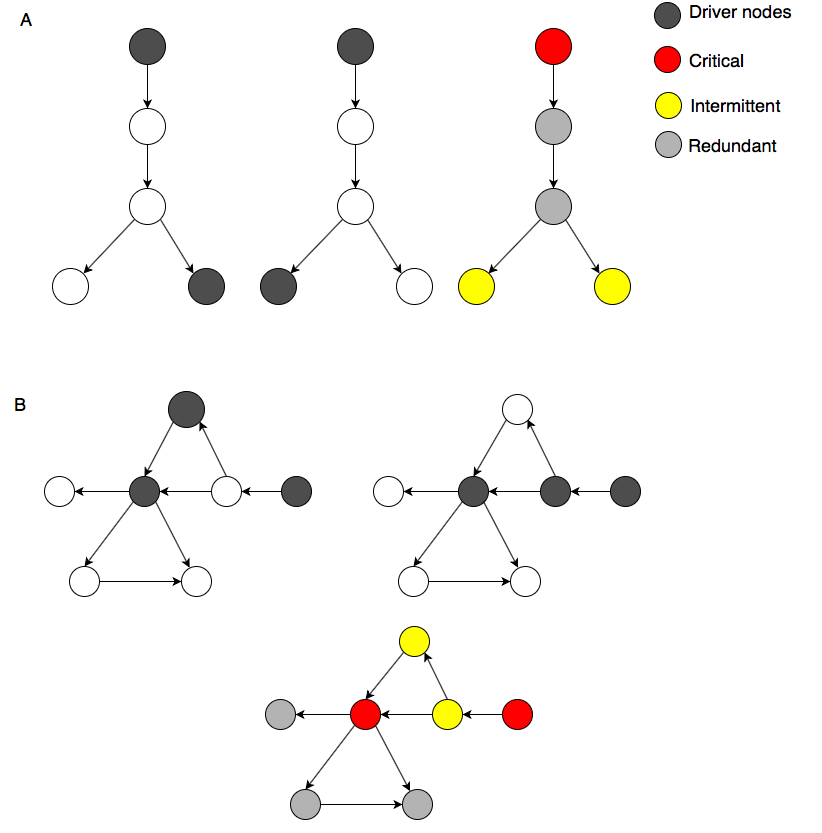

Supplement: Supplementary file 2 — LaTeX Supplementary file [file 41598_2018_38224_MOESM2_ESM.zip › MDS_model_2.png]
